# Reply to the EFSA (2016) on the relevance of recent publications (Hofmann et al. 2014, 2016) on environmental risk assessment and management of Bt-maize events (MON810, Bt11 and 1507)

**DOI:** 10.1186/s12302-017-0106-0

**Published:** 2017-03-07

**Authors:** Maren Kruse-Plass, Frieder Hofmann, Ulrike Kuhn, Mathias Otto, Ulrich Schlechtriemen, Boris Schröder, Rudolf Vögel, Werner Wosniok

**Affiliations:** 1 TIEM Integrated Environmental Monitoring, Dortmund/Bremen, Germany; 2 Wölsauerhammer, Marktredwitz, Germany; 3 Ökologiebüro, Bremen, Germany; 4 Büro Kuhn, Bremen, Germany; 5 Federal Agency for Nature Conservation (BfN), Bonn, Germany; 6 Sachverständigenbüro, Dortmund, Germany; 7 Landscape Ecology and Environmental Systems Analysis, Institute of Geoecology, Technische Universität, Brunswick, Germany; 8 Berlin-Brandenburg Institute of Advanced Biodiversity Research (BBIB), Berlin, Germany; 9 Agency for Environment, Health and Consumer Protection, Eberswalde, Brandenburg Germany; 10 Institute of Statistics, University of Bremen, Bremen, Germany

**Keywords:** Genetically modified organisms, Environmental risk assessment, Exposure, Host plants, Non-target organisms, Lepidoptera, Pollen deposition, Isolation buffer zones

## Abstract

In this commentary, we respond to a report of the EFSA GMO Panel (EFSA EFSA Supp Publ, 1) that criticises the outcomes of two studies published in this journal (Hofmann et al. Environ Sci Eur 26: 24, 2; Environ Sci Eur 28: 14, 3). Both publications relate to the environmental risk assessment and management of Bt-maize, including maize events MON810, Bt11 and maize 1507. The results of Hofmann et al. (Environ Sci Eur 26: 24, 2), using standardised pollen mass filter deposition measurements, indicated that the EFSA Panel model had underestimated pollen deposition and, hence, exposure of non-target organisms to Bt-maize pollen. The results implied a need for safety buffer distances in the kilometre range for protected nature reserve areas instead of the 20–30 m range recommended by the EFSA Panel. As a result, the EFSA Panel revised their model (EFSA EFSA J 13: 4127, 4), adopting the slope of the empirical data from Hofmann et al. The intercept, however, was substantially reduced to less than 1% at one point by introducing further assumptions based on the estimates of mainly panel members, citing possible ‘uncertainty’. Hofmann et al. (Environ Sci Eur 28: 14, 3) published extensive empirical data regarding pollen deposition on leaves. These results were part of a larger 3-year study involving detailed measurements of pollen release, dispersal and deposition over the maize flowering period. The data collected in situ confirmed the previous predictions of Hofmann et al. (Environ Sci Eur 26: 24, 2). Mean levels and observed variability of pollen deposition on maize and four lepidopteran host plants exceeded the assumptions and disagreed with the conclusions of the EFSA Panel. The EFSA Panel reacted in a report (EFSA EFSA Supp Publ, 1) criticising the methods and outcomes of the two published studies of Hofmann et al. while reaffirming their original recommendations. We respond here point-by-point, showing that the critique is not justified. Based on our results on *Urtica* leaf pollen density, we confirm the need for specific environmental impact assessments for Bt-maize cultivation with respect to protected habitats within isolation buffer distances in the kilometre range.

**Electronic supplementary material:**

The online version of this article (doi:10.1186/s12302-017-0106-0) contains supplementary material, which is available to authorized users.

## Background

In this commentary, we respond to a report of the GMO Panel of the European Food Safety Authority (EFSA) in 2016 [1] that criticises the outcomes of two of Hofmann et al.’s studies published in this journal in 2014 and 2016 [2, 3]. Both publications relate to the environmental risk assessment and management of Bt-maize in the EU [4], including maize events MON810, Bt11 and maize 1507.

Evaluating non-target effects is part of the environmental risk assessment process prior to release and commercial use of genetically modified organisms (GMOs) as prescribed in EU regulations [5]. Bt-maize shares transgenes in its DNA that are derived from the soil bacterium *Bacillus thuringiensis*, which enables the plant to express insecticidal toxins [6, 7]. While protecting the plant against target herbivores, these toxins are also known to negatively affect non-target organisms such as Lepidoptera [8]. Because Bt toxins are expressed in all tissues of the plant, they are also dispersed into the environment by pollen. This leads to exposure to the toxin by non-target organisms, such as Lepidoptera larvae, which feed on host plant leaves.

In their initial environmental risk assessment regarding Bt-maize cultivation, the EFSA Panel used a mathematical model to form their opinions [4, 9–13]. The model was developed by members of the GMO Panel at the time [14–17] and has been used in regulatory risk assessments since 2009 [9]. The model consists of two combined equations: a dose–distance relationship and a dose–mortality relationship for five hypothetical species feeding on nettle (*Urtica diocia)* leaves. The model was the first approach to this problem and used a limited dataset—both for exposure and effects—and thus required a series of assumptions [8, 18]. The dose–distance relationship, for example, was based until 2015 on a small dataset on pollen deposition derived from exposure of slides near to a single maize field for 7 days [19]. Assumptions were made to transfer the pollen deposition measurements from this technical sampling to pollen density on *Urtica* leaves. This base led the EFSA Panel to recommend isolation buffer distances of 20–30 m as being sufficient to protect sensitive non-target Lepidoptera in cultivation areas of Bt-maize events MON810, Bt 11 and 1507. The EFSA Panel regarded their calculations as overestimating exposure risks and effects, reflecting a worst-case scenario [4, 14].

Hofmann et al. [2] analysed a representative dataset on maize pollen deposition measurements collected using a standardised method at 214 sites over 10 years from inside maize fields to 4.42 km from the nearest maize pollen source. The results significantly exceeded the deposition estimates made by the EFSA GMO Panel for distances greater than 10 m [9–13]. The EFSA model, which relied on extrapolation of observations that were limited to a distance of up to 7 m from field edges, resulted in a much steeper negative slope compared with measured deposition, as illustrated in Fig. 5 of [2] (see Additional file 1: Figure S1). Based on their empirical results, Hofmann et al. [2] confirmed the findings of previous studies [20–24] that isolation buffer distances in the kilometre range are likely to be required to maintain the threshold limits calculated by the EFSA Panel for effective protection of sensitive Lepidoptera. In addition to other impacts, these results also affected legislation in Germany.

The German Advisory Council on the Environment (SRU) ([25], chapter 12.3.3) recommended isolation buffer distances of at least 1000 m from protected habitats. The SRU has demanded that an environmental impact assessment be carried out regarding specific local conditions when Bt-maize cultivation is planned within the buffer distance. Isolation buffer distances in the range of 800–3000 m from protected habitats have been implemented in some German States (e.g. Brandenburg, Baden-Württemberg, Bavaria, Saxony). Outside of the isolation buffer zone, cultivation is permitted.

The EFSA Panel published a revised risk model [4] to incorporate the new data of Hofmann et al. [2]. Instead of adapting their model to the published empirical deposition data, the EFSA Panel decided to incorporate the slope of the distance relationship in [2] but to reduce the intercept by several factors to lower the exposure estimate. In this work, the EFSA Panel classified the estimates of previous studies [9–12] as being ‘unrealistically high’. Thus, the Panel introduced a whole set of new uncertainty factors to adjust the exposure estimate to a ‘realistic’ level.

The EFSA Panel calculated three scenarios (Table 1 [4]):

The ‘direct comparison’ (DC) scenario divided the original data by 4 (factor 0.25) based on the argument that it was necessary to make the empirical data ‘compatible’ with the EFSA exposure model, which was based on a shorter flowering and exposure period (e.g. 7 days instead of average 4 weeks). The justification for the original shorter period in the EFSA model was that >92% of pollen shed [17] would take place within the first 7 days.

For the ‘most realistic’ (MR) scenario, the EFSA Panel determined 8 uncertainty factors using averaged estimates decided by the panel members (see Additional file 1: Table S2). The resulting uncertainty factor of 0.0376 was applied in addition to the DC factor to reduce the measured deposition data from the standardised technical sampling to *0.94%* (0.25 × 0.0376 = 0.0094 = 0.94%) of initial values.

A third ‘conservative’ (CO) scenario was calculated to simulate the worst case (1 in 40 cases) for protection of highly sensitive species in nature reserve areas. Here, a factor of 9 was employed to reflect the variation between sites based on the pollen mass filter (PMF) measurements [20]. However, the variation within sites (e.g. between plants, leaves and within leaf surfaces), was not included. The EFSA Panel applied this factor to their reduced `most realistic´ MR dataset, and not to the original data. This led to a combined reduction factor of 0.0846 being applied to the *mean* dose–distance relationship of the data [2] to determine the worst-case scenario.

In addition, the EFSA Panel introduced a hypothetical 1 × 1 km^2^ nature reserve area to their revised model. Exposure and mortality effects were averaged within this model nature reserve by calculating weighted averages over three distances (for an adjacent nature reserve: 5, 500, 1000 m). Thus, the estimates of exposure and effects were further diminished.

After revising their model, the EFSA Panel concluded [4] that no changes to the previously proposed 20–30 m buffer between Bt-maize cultivation and protected areas would be required under ‘realistic’ or even ‘worst case’ assumptions.

In contrast to the EFSA Panel’s approach, an integrated 3-year assessment in the region of the Biosphärenreservat Schorfheide-Chorin nature reserve in Brandenburg, Germany [3, 26, 27] covered the whole process of pollen exposure, including the configuration of maize fields and nature reserve areas within a 1250 km^2^ region (35 km × 35 km). The study provided a comprehensive dataset based on standardised measurements and included meteorological data, pollen release rates and aerial concentrations inside and outside of selected maize fields. The work modelled pollen dispersal for all maize fields in the area and evaluated pollen deposition using standardised technical sampling as well as direct measurements of plant leaves. In 2010, a total of 5377 measurements of pollen density on the leaves of maize and four lepidopteran host plant species inside and outside maize fields were collected over the whole flowering period. The integrated assessment enabled the determination of relationships between pollen release rates, aerial concentrations and deposition on the PMF samplers according to national (German VDI Standard 4330-3 [28]) and European (CEN-TS 16817-1 [29]) standards with plant-specific leaf pollen density. By calibrating the plant-specific leaf deposition data to the standardised deposition measurements, a distance relationship for pollen deposition on host plant leaves was determined. This relationship was used to estimate the amount and variability in the pollen density on the leaves of maize, *Urtica* and three other host plants in the range from 0.2 m to the next pollen source (within the field) to 4.42 km away under commercial cultivation conditions.

These field-based data are in line with results of model scenarios [22, 30–32]. The studies regarded realistic complex field configurations under commercial cultivation and they showed the overlapping exposure and effects with an enlarged tail in the distance relationship well beyond the 20–30 m range in contrast to the isolated single-field approach in the EFSA Panel risk assessment.

The EFSA Panel [1] reacted to the findings of Hofmann et al. [3] by ignoring the relevance of these empirically based results and stated that *“EFSA considers that the previous risk assessment conclusions and risk management recommendations on maize MON810, Bt11 and 1507 for cultivation made by the Panel on Genetically Modified Organisms remain valid and applicable”.* The EFSA Panel justified this opinion based on supposed methodological errors in [3].

In this commentary, we respond point-by-point to the EFSA Panel opinion [1] and demonstrate that their critique is not substantiated. As a basis for this discussion, we compare the estimates of the EFSA model scenarios on the dose–distance relationship for leaf pollen density on *Urtica* with the empirically based findings of [2, 3]. Finally, we discuss the implications for risk assessment and management with regard to sensitive butterfly species in protected habitats.

This commentary focuses on the aspects of pollen dispersal and deposition as part of the exposure model in the EFSA Panel ERA model [4]. For a critical discussion of other assumptions employed by the EFSA Panel model concerning the variation in Bt-concentration and the sensitivity of species to this toxin as well as the dose–effect relationship and the subsequent potential underestimation of effects we refer to [8, 18, 33, 34].

## Comparing the EFSA scenarios for the dose–distance relationship of *Urtica* leaf pollen density with the empirical findings of Hofmann et al.

**Fig. 1:**
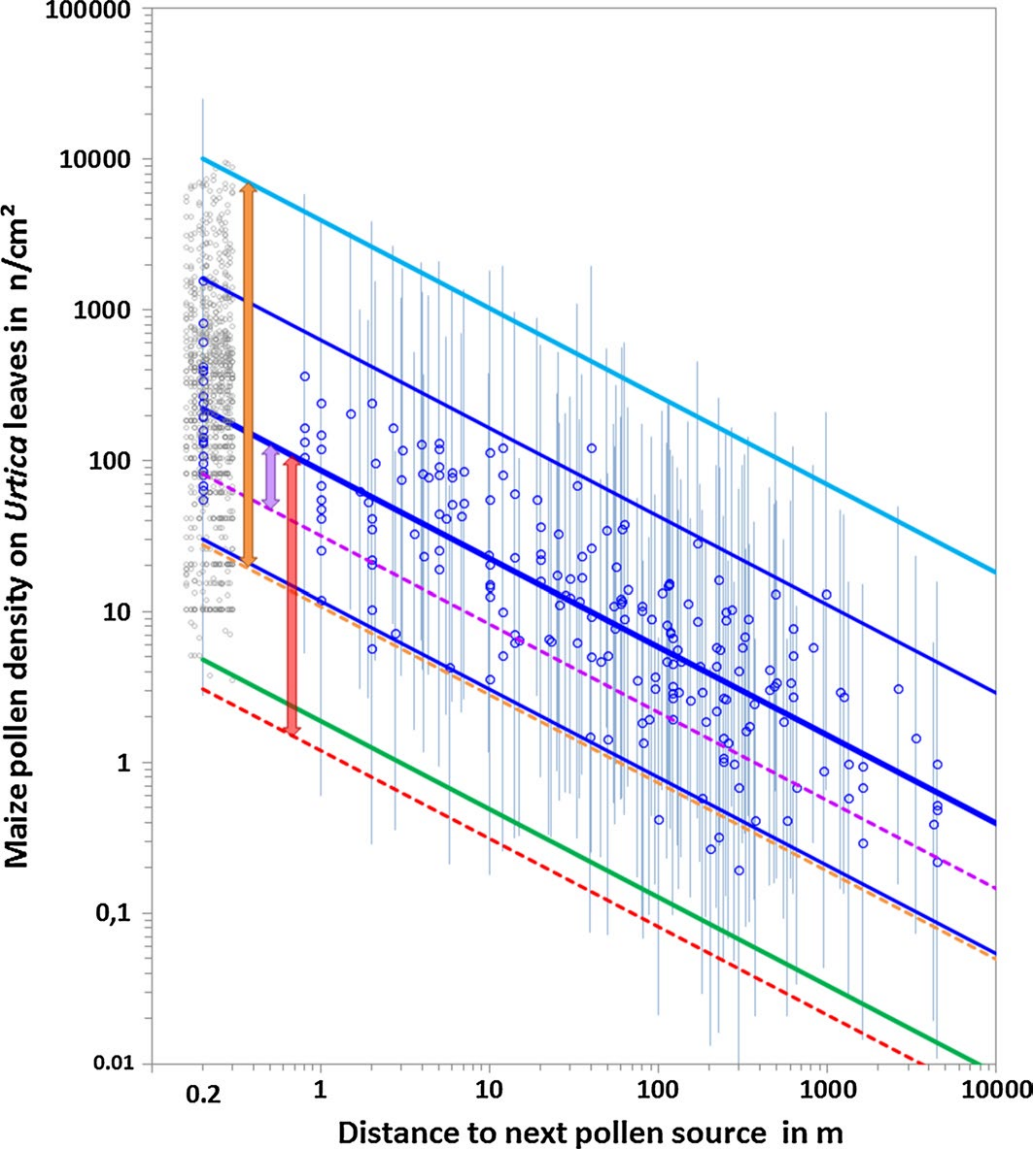
Variability of *Urtica* leaf pollen density over distance. Comparison between the three scenarios of the EFSA Panel model [4] and the empirically based findings of Hofmann et al. [3].  RS ‘realistic’ mean dose–distance relationship leaf pollen density Urtica [3];  WC ‘worst case’ scenario upper 95% CI leaf density data [3];  lower 95% CI leaf density data [3];  mean leaf pollen density* Urtica* per site during flowering period by standardized and calibrated PMF measurements (*n* = 214) with 95% confidence interval for single values [3];  95% CI for mean leaf pollen density* Urtica* per site per standardized and calibrated PMF measurements [3];  leaf pollen density data* Urtica* close to the pollen source indicating the variability and used for calibration (*n* = 836 measurement data, scattered around 0.2 m distance for displaying the variability close to source) [3];  DC—‘direct comparision’ scenario EFSA panel model 2015 [4];  MR—‘most realistic’ EFSA 2015 model [4];  CO—‘conservative’ EFSA 2015 model worst case 1:40 [4];  Difference between ‘MR—most realistic’ scenario EFSA 2015 [4] and ‘RS—realistic mean’ regression [3]: ratio 0.0138;  Difference between ‘CO—conservative’ scenario EFSA 2015 [4] for 1:40 worst case and respective ‘WC—worst case’ [3]: ratio 0.00273;  Difference between ‘DC—direct comparison’ EFSA 2015 [4] and ‘RS—realistic mean’ regression [3] based on measurement data: ratio 0.368

Figure 1 compares the outcomes of the EFSA Panel model [4] with the empirically based results of Hofmann et al. [2, 3] on the dose–distance relationship for *Urtica* leaf pollen density. The blue circles indicate the results of [2, 3] showing the variability of mean pollen density on *Urtica* leaves during the flowering period for each site over the distance range derived from the standardised PMF pollen deposition data (*n* = 216) calibrated to *Urtica* leaf pollen density by in situ measurements close to the pollen source (*n* = 834). Grey circles indicate the results for the variability of single density values close to the source, and 95% confidence intervals (blue vertical bars) show those for the mean density values at each site.

For all following equations, *d* denotes leaf pollen density in n/cm^2^ and *S* represents distance in m.

The findings of the regression analysis in Hofmann et al. [3] based on empirical data are as follows:

For the ‘realistic scenario’ (RS), the mean regression with a 95% confidence interval is taken according to Eq. 1 (below), indicating the variability of leaf pollen density values for *Urtica* over a distance range of 0.2 m from the pollen source (within the field) to 4.42 km away under common cultivation conditions (solid blue line in Fig. 1):

1 $${\text{RS}}{:}\;d_{\text{RS}} = 8 6. 2S^{ -0.585}\quad \text{or} \quad\text{log}_{10} d_{\text{RS}} = 1.935 - 0.585 {\text{log}}_{10} S$$

$$\sigma = 0.887 { }\left( {{ \log }_{10} {\text{scale}}} \right);{95}\% \;{\text{CI mean}} \pm 1.774 { }\left( {{ \log }_{10} \;{\text{scale}}} \right)$$

The variability of mean leaf pollen density is denoted by the 95% confidence interval for the mean regression curve. Figure 1 displays the 95% confidence interval for the mean density values for each site and flowering period (thin blue lines around the mean) and the 95% confidence values for single leaf density values with a factor of 48 around the mean on a normal scale (green and cyan lines). The corresponding dose–distance relationship indicating the ‘worst case’ scenario (1 in 40 cases; WC) is given by the upper 95% confidence boundary for leaf density values (cyan line) by Eq. 2:

2 $${\text{WC}}{:}\;d_{\text{WC}} = 3949S^{ - 0.585} \quad\text{or} \quad\text{log}_{10} d_{\text{WC}} = 3.596- 0.585 {\text{log}}_{10} S.$$

The three scenarios of the EFSA Panel model (DC, MR, CO) are given in Table 1 [4] as follows:

The ‘direct comparison’ (DC) to deposition on technical samplers follows Eq. 1 and is shown by a dotted pink line in Fig. 1:

3 $${\text{DC}}{:}\;d_{\text{DC}} = 31.8S^{ - 0.585}.$$

The ‘most realistic’ scenario (MR) follows Eq. 2 and is given as a dotted red line in Fig. 1:

4 $${\text{MR}}{:}\;d_{\text{MR}} = 0.0376d_{\text{DC}} = 1.20S^{ - 0.585}.$$

The ‘conservative’ scenario (CO) indicating a worst-case situation (1 in 40 cases) follows Eq. 3 and is shown by a dotted orange line in Fig. 1:

5 $${\text{CO }}\left( {{\text{worst case 1:}}40 \, 95\% \;{\text{CI}}}\right){:}\; \, d_{\text{CO}} = 9d_{\text{MR}} = 10. 8S^{ -0.585}.$$

The comparison in Fig. 1 shows that all three EFSA model scenarios [1, 4] underestimate the variability and intensity of leaf pollen deposition over the whole dose–distance relationship (from close to the source within a field to 4.42 km away) compared with our results based on realistic and worst-case estimates. The ratio between the MR scenario of the EFSA Panel and the ‘realistic’ mean regression of pollen density on *Urtica* leaves based on the deposition measurements in [2, 3] is 0.0138 (difference indicated by the red arrow in Fig. 1). The ratio between the DC scenario of the EFSA Panel and the RS based on [2, 3] is 0.368 (difference indicated by the pink arrow in Fig. 1). For the worst-case scenario, the ratio between the CO of the EFSA Panel and the WC based on the results of [2, 3] is 0.00273 (difference indicated by the orange arrow in Fig. 1).

**Fig. 2:**
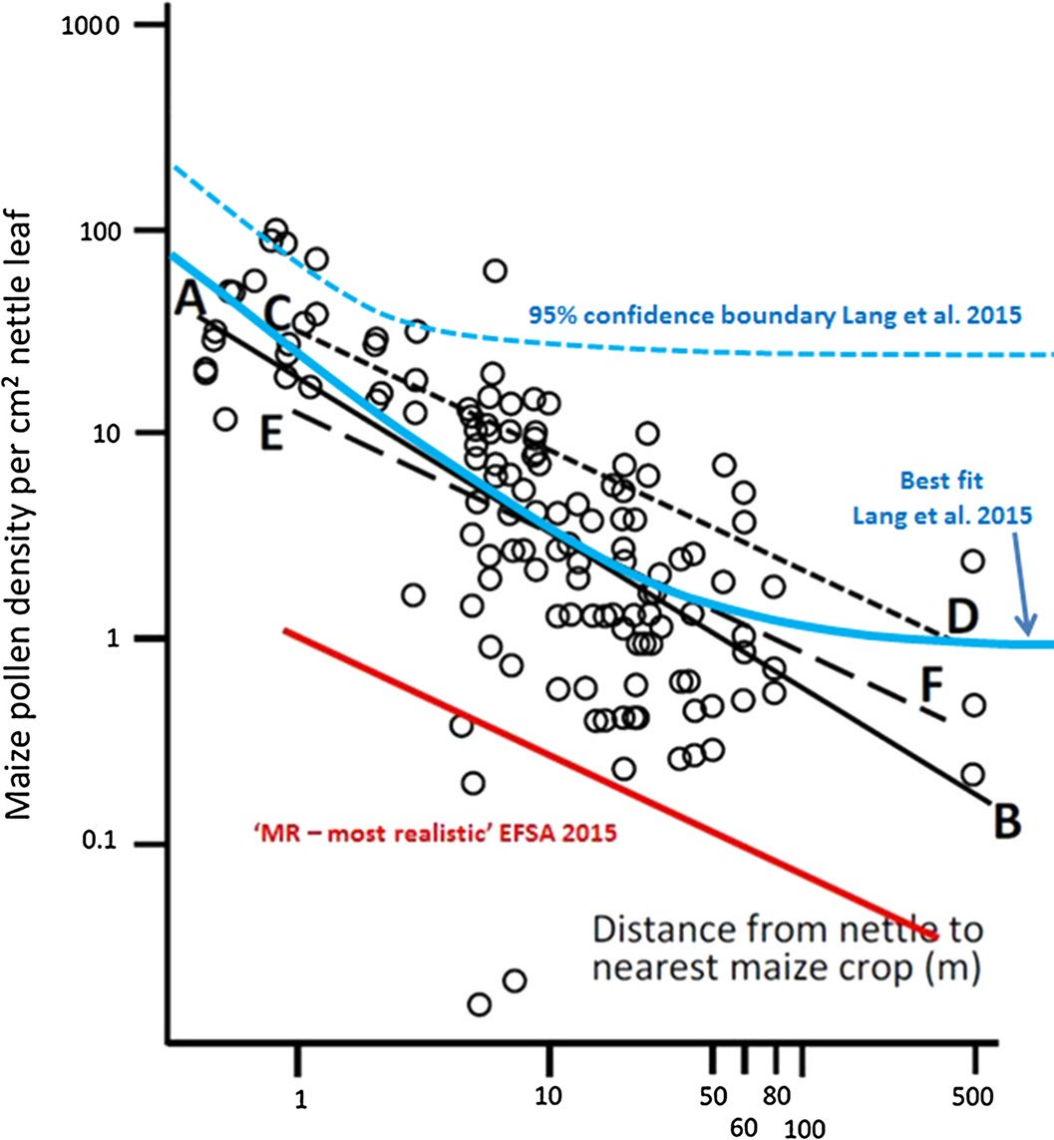
Dose–distance relationship in Fig. 1, EFSA [1]. “*Digitised data from Fig. 3 of Lang et al. (2015)* [35] *relating dose of pollen counted per cm* ^*2*^ *of nettle leaf to the distance of the nettle plant from the edge of the nearest maize crop plant. Data plotted on double logarithmic axes to match scaling adopted by Hofmann et al. (2014)* [2]*. Lines shown on figure are AB: regression line of best fit; CD: dose–distance relationship of Hofmann et al. (2014)* [2] *as used in scenario DC of EFSA (2015)* [4]*; EF: dose-distance-relationship of Hofmann et  al. (2014)* [2] *reduced by multiplicative product, 0.396, of two exposure factors estimated in EFSA (2015)* [4]*, corresponding to scenario MR.*” EFSA [1]. The original dose–distance relationship for the ‘most realistic’ scenario in EFSA [4] has been calculated using a factor of 0.0376 and not 0.396 as stated in EFSA 2016 [1]. The correct line (*red line*) is given here as approximately ten times lower than the level given in EFSA [1]; (*EF line*). Further, we included the 95% confidence boundaries mentioned by Lang et al. [35]. Both the corrected *line* and the confidence boundaries from Lang et al.’s study demonstrate the underestimation of exposure risk in the EFSA Panel’s ‘most realistic’ scenario

We further note that in Fig. 1 in the EFSA Panel’s report [1], the dose–distance relationship ‘EF’ does not relate to the ‘most realistic’ scenario of the EFSA Panel [4], although it is implied to do so. The factor applied (0.396) is different from that of the original MR scenario in [4] (0.0376). The EF line for the EFSA Panel MR scenario should correctly be 10 times lower and would thus lie below the leaf density data of Lang et al. [35] as shown in Fig. 2.

Furthermore, data of Lang et al. [35] agree well with our assessment as these results depict mean leaf density measurements on single days at each site in one region without representing the variability of leaf density values over time and space under common cultivation. Their confidence limits, again, correspond well with our results and confirm the extent of variation to be expected.

## Reply to EFSA Panel [1]

In the following, we respond point-by-point to the EFSA Panel’s critique [1] of the methods and findings of Hofmann et al. [2, 3].

### Relationship of standardised pollen deposition to leaf pollen density

The EFSA acknowledges the value of the standardised method as the basis for data collection but argues that the *“most important gap in the information in Hofmann et al.* [2] *is that there are no data to enable any calibration between pollen density measured by the mechanical sampler and the pollen density per cm* ^*2*^ *leaf surface as encountered on a host plant by a NT lepidopteran larva at the same spatial location”.*

Hofmann et al. [2] focused on the distance relationship in pollen deposition as measured by the PMF standardised technical sampling method. The calibration of the standardised pollen deposition measurements to plant-specific leaf pollen density per cm^2^ for maize, *Urtica* and three other Lepidoptera host plants has been presented in detail in [3] and the corresponding reports on the research project [26, 27].

As explained above (see the “Background” section), the experiments analysed in [3] included an integrated assessment of pollen release rates, aerial pollen concentrations and pollen deposition measurements at the same location, which enabled calibration between standardised pollen deposition data and leaf pollen density data collected through in situ measurements in the field.

In contrast, the EFSA Panel based its estimate of *Urtica* leaf pollen density on the model in [14], which was developed using a small dataset of pollen deposited on Vaseline-coated slides collected in the US [19]. The EFSA Panel further divided the results by a factor of 3 based on studies in the US [36] and Germany [37] stating that slides probably overestimate leaf deposition. Further, the EFSA Panel assumed a factor of 2.85 based on data from Hungary [38] to infer to *Urtica* leaf pollen density. However, these studies have little in common. All use different methods, present data from different sites, regions and years, and are not standardised to allow either direct comparison or meaningful combination in a single model.

In the EFSA Panel’s revised model approach [1, 4] the base of exposure was modified to incorporate the PMF data. However, data were modified to compensate for assumed uncertainties; the EFSA Panel applied 8 additional factors based on their average expert estimates that led to a combined reduction factor of 0.0094 as explained above (see also Additional file 1: Table S2; Table 1 [4]). This meant that the additional factors actually decreased the assumed pollen deposition values to lower than the original model assumptions. The relationship factor between PMF pollen deposition data and the mean leaf pollen deposition according to the EFSA Panel is thus 73 times lower than the factor of 0.68 that was based on field measurements, as documented in [3] (see Fig. 1). The inherent uncertainty of the EFSA Panel’s approach is reflected in the exposure assessment and may explain the discrepancies between the EFSA model estimates of the dose–distance relationship and our empirically based findings.

### Consistency of the dose–distance relationship

The EFSA Panel argues that the *“dose*–*distance relationship, derived by Hofmann et al.* [2] *from regression on logarithmic scales, should have been based on their data from samplers placed at distances from the nearest maize crop ranging from 0.8* *m to 4.4* *km only. However, Hofmann* et al. [2] *also included samples taken from within the crop which, in the context of their regression analysis, were assigned, a negative value as distance from the crop edge. Consequently, because logarithms of negative numbers do not exist, such data cannot validly be used to contribute towards the calculation of the regression relationship on logarithmic scales. Hofmann* et al. [2] *wrongly included these withincrop data in their regression calculations. EFSA* [4] *did not highlight this limitation, because for protected habitats, at distances of the order of more than tens of metres from a maize field, the inclusion or exclusion of a few data points from within the maize crop would make little difference to the relationship derived. However, it must be emphasised that in the Hofmann* et al. [2] *publication there is no valid published information concerning the relationship between pollen deposition and distance from within the Bt*-*maize crop or at any distance up to 0.8* *m from the edge of the crop”.*

Any dispersal of pollen starts from a maize plant as the pollen source, with dispersal distances being greater than zero. In our study, we took 0.2 m as the closest distance from the target plant to the next maize plant (the pollen source), which is incorporated in the within-field data. The results of the statistical analysis in ([2], Figure 3) (see Additional file 1: S3) clearly show that a distance of 0.2 m is an appropriate starting value for the regression analysis. The results gave a consistent dose–distance relationship over the entire distance range: from 0.2 m from the pollen source inside the field to 4.42 km away, as shown in the statistical analysis in [2], Figures 3 and 4 (see Additional file 1: S3, S4). Owing to the variability in field shapes and configurations, this was the best way to describe the variation in pollen deposition inside and at the edge of maize fields. In contrast, the EFSA Panel model uses negative distance values for inside-field sites and this trend continues with a sudden drop in deposition levels at field edges (zero distance). In reality, there cannot be a negative distance to a maize plant as a pollen source, which leads to inconsistencies. Furthermore, the EFSA Panel’s split-level model with its sudden drop in deposition values at the field edge (distance 0 m) is not grounded in reality. Instead, the variability in pollen deposition is high both within fields and at field edges, with overlapping values; indeed, the decrease in deposition is represented by a smooth curve [39, 40], as seen in our field data.

### Supporting information

The EFSA argues that *“the data published in Hofmann et al.* [2] *recorded the distance to the nearest maize crop, but gave no information regarding the number of maize fields in the area contributing to pollen deposition, or their location, or maize variety”.*

Isolation buffer distances according to the EFSA Panel’s recommendations only consider distance to the next maize field. No other information is taken into account. This constraint is reflected representatively by the Hofmann et al. study [2].

The strength of the Hofmann et al. data lies in the wide range of conditions encountered over 10 years of collecting field data collection at different sites and regions. These data represent the variability of deposition under common cultivation conditions including different field sizes and configurations, varieties, regions and environmental conditions and relative positions of the measurement site to the field with respect to wind direction. Hofmann et al. [2] analysed data from more than 20 research and monitoring projects at 214 sites. The Additional file 1 with detailed data on the maize field configurations, meteorology and other background information can be found in the reports of the respective projects, which are cited in Table 1 of [2].

### Sampling method

The EFSA postulated that the method Hofmann et al. used for estimating the density of pollen on individual leaves did *“not employ random sampling, but is designed to deliberately include areas of high pollen density on leaves, resulting in statistically biased, overestimates of pollen deposition”.*

The EFSA argument is based on an incorrect interpretation of the sampling method. Random sampling is not the only way of obtaining unbiased samples. Sometimes other methods perform better for statistical reasons (theoretically shown in Section 11.4 of [41]). The method used for sampling in situ pollen leaf deposition has been validated and is published in an international peer-reviewed scientific journal [42]. The method relies on a stratified sampling design. It was developed after four different sampling designs had been evaluated. Random sampling was one of the four methods tested, but it failed to sufficiently represent the high variability of leaf deposition data. The combined structured design resulted in 27 samples (microscopic fields) per leaf consisting of three transects with five samples and two clusters in areas of high and low pollen density with four samples each. This method best depicted the variability of pollen deposition on plant leaves in terms of mean, variance and peak values of the true total variability of leaf pollen density values, as shown in Table 1 of [42] (see Additional file 1: S5; column ‘Total’). Hence, the results of the method were balanced and not biased.

Furthermore, three leaves from three plants were counted for each site and date, resulting in 81 measurements. The measurements were repeated on the same leaves and plants and in the same areas defined at the beginning of the study covering the whole flowering period. These comprehensive measurements allowed for the detailed assessment of variability in leaf pollen deposition in detail for each site over time. Based on a statistically validated sampling design, these data represent the most detailed leaf deposition measurements reported in the literature so far. The results also indicated that mean leaf measurements of single plants or days were not representative and that leaf pollen deposition over the flowering period must be considered. Furthermore, a detailed statistical analysis of the data distribution clearly shows that there was no bias in the data distribution ([3] Figure 4, see Additional file 1: S6).

In contrast, the data of the EFSA Panel [1, 4] relied on estimates derived mostly from single or a few measurements, reflecting mean leaf pollen density values of single leaves, plants or days only. Measurements covering variation over the entire flowering period are not included. For example, the data from Lang et al. [35], cited in the EFSA Panel [1], were based on single measurements of mean leaf pollen density taken only once during flowering at each site.

### Standardised methods and data

EFSA Panel criticises the Hofmann et al. data, stating, “*all the data from within the Bt*-*maize crop and at crop edges were standardised to relate to the same distance from the pollen source. The relationship used for the standardisation was the Hofmann et al.* [2] *dose*–*distance relationship for distances from 0.8* *m to 4.45* *km, but, as explained in Section* *3.1.2, above, this cannot be used for data within the maize crop and at crop edges. Hence, all data have been standardised, involving potential multiplication or division by five fold or more, using a relationship with no evidential basis for the data on which it is used. The standardisation is unnecessary; information should be given which facilitates identification of the relationship between pollen deposition dose and distance, as in Perry et al.* [17]*, Hofmann et al.* [2] *and Lang et al.* [35]*)”.*

Leaf pollen density data vary considerably between days, within days, between sites and within sites, between plants, between leaves and on the leaf surface [3, 26, 43]. Thus, methods and data must be standardised to achieve comparable results. Furthermore, an appropriate method must be used for each task. To assess the variability of pollen deposition between sites and over distances, standardised methods are required, such as the PMF [2, 28, 29]. To assess the variability of leaf pollen density values within a site, between plants and leaves and on the leaf surface, direct in situ measurements are appropriate [3, 42].

The distance relationship for *Urtica* leaf pollen deposition was based on standardised measurements using the PMF at 214 sites covering a distance range from 0.2 m to the next pollen source inside a field to up to 4.42 km away. These measurements were calibrated to *Urtica* leaf density by 836 in situ measurements close to the source. The results provide a consistent dose–distance relationship for the whole distance range. In contrast to the EFSA Panel’s statement, no breaks, further standardisation, or multiplication of data were applied in the statistical analysis of the distance relationship for pollen deposition on *Urtica* leaves. Pollen deposition values were calibrated by parallel measurements at the same site, which validates the calibration because the dose–distance relationship is based on consistently observed data. Furthermore, the results at the same site were supported by standardised technical measurements of pollen release rates continuously recorded by a volumetric pollen monitor with omnidirectional inlet (PMO). The data from the pollen monitor represented the temporal changes and intensity of pollen shedding over the flowering period. The results regarding the dose–distance relationship for *Urtica* leaf pollen deposition showing variability over distance are displayed in Fig. 1.

The data from Lang et al. [35] cited by the EFSA Panel [1] relied on single measurements of mean leaf density values per site by washing pollen off of single leaves. The measurements were taken on different days with no measure of the intensity of pollen shedding or deposition at the site. Therefore, the data do not represent the variability in leaf pollen density over space and time as illustrated by our measurements over the flowering period (see Fig. 1 and [3]; Figures 3 and 4, Additional file 1: S6, S7). Thus the methods of Lang et al. give no representative data for comparison of the results between sites: Further, standardised deposition measurements were not applied. Thus, unfortunately, the results of that study cannot be generalised. However, the data lie within our confidence boundaries, so they do not contradict our more representative results.

### Exposure of protected habitats

The EFSA Panel argues: * “Hofmann et al.* [3] *reported no data on pollen deposition outside maize fields; in particular, no data are presented that would enable verification of pollen deposition assumptions on host plants found in protected habitats”.*

As documented in Hofmann et al. [3], pollen deposition was measured inside and outside of maize fields, which included nature reserve areas. The study area encompasses a region with rich biological diversity that is well known for its nature reserve areas [26, 27]. Further, considerable maize pollen deposition inside protected areas, and particularly on the leaves of protected butterfly host plants, was found in previous studies [23, 24, 44] concerning the region of the Ruhlsdorfer Bruch nature reserve area in Brandenburg, an area listed according to the Fauna–Flora-Habitats-Directive (FFH) and inhabited by protected Lepidoptera.

During sampling in those studies, Bt-maize was grown in the vicinity of the study area and transgenic Bt-maize pollen was positively detected in several sites covering the nature reserve area. Up to distances of 250 m from the next maize field, the furthest measuring point, Bt-maize pollen was identified using polymerase chain reaction methods (PCR) on PMF samples taken in the centre of the nature reserve area. These results were supported by detection of Bt-maize pollen on the leaves of various Lepidopteran host plants.

Border rows of 20 m had been used by the farmers in the area as a buffer to the protected area. These measurements are therefore particularly relevant for evaluating the EFSA Panel’s proposed mitigation measure of 20–30 m buffer areas.

Thus, the results from the Ruhlsdorfer Bruch study demonstrate that the buffer distances proposed by the EFSA Panel would have been insufficient to prevent protected butterfly species in nature reserve areas from maize pollen exposure. In fact, Bt-maize pollen deposition and maize pollen densities on leaves up to 3000 n/cm^2^ were detected within the protected area. One possible reason for these high pollen densities is that maize pollen can be lifted by thermal gusts, which occur particularly in the summer on bright and windy days. These conditions also encourage maize pollen release and dispersal by wind to farther distances [22, 26, 45–48].

### No need for model revision?

The EFSA assumes that it *“…is generally agreed that pollen deposition declines with increasing distance from the nearest pollen source, but proposed relationships governing this decline differ (see review by Perry et al.* [17]*). In particular, for the GMO Panel Scientific Opinions published prior to 2014* [9–13] *the assumed dose of pollen on host plants within the Bt*-*maize source crop was almost seven times greater than that assumed under the relationship for mechanical samplers published by Hofmann et al.* [2]*. Furthermore, outside the source crop, the assumed dose was greater than that assumed by Hofmann et al.* [2] *up to 9* *m from the crop edge. In contrast, beyond 9* *m from the edge of the crop, and in particular at distances greater than 30* *m, the dose assumed by Hofmann et al.* [2] *was much larger than that assumed in GMO Panel Scientific Opinions. Since within and close to the Bt*-*maize field the estimates of mortality made by the GMO Panel* [9–13] *exceed those that would be derived assuming the Hofmann* et al. [2] *relationship, there was no need to revise the consequences for the previous EFSA risk assessment conclusions and risk management recommendations for Bt*-*maize for NT larvae within the field and its margins”.*

The recommendations of the EFSA Panel prior to 2015 [9–13] based their exposure estimates on the model of Perry et al. [14–17], which takes the mean pollen density of *Urtica* leaves as cumulative 7-day deposition values of 589.9 n/cm^2^ within the maize source crop and 221.8 n/cm^2^ at the field edge. Perry et al.’s model relies on the assumption that >92% of total pollen deposition during the flowering period occurs in the first 7 days [17]. Thus, the mean pollen density on *Urtica* leaves per day for the whole flowering period was calculated as 85.56 n/cm^2^ within the field and 31.69 n/cm^2^ at the field edge (see [17], Table A1, at the bottom of the table). The in situ measurements within-field pollen density on *Urtica* leaves by Hofmann et al. [3] demonstrated density values up to 13,802 n/cm^2^ and mean pollen density values up to 2710 n/cm^2^. That is, the empirical values were more than four times greater than those estimated by the EFSA Panel (pollen density in the field according to [3] 2710 n/cm^2^ divided by leaf density in the field according to [15] 589.9 n/cm^2^ = 4.5). Furthermore, mean daily pollen density on *Urtica* leaves was 244 n/cm^2^, which is 2.8 times higher than the EFSA Panel’s estimate (mean daily pollen density according to [3] 244 n/cm^2^ divided by leaf density according to [17] 85.56 n/cm^2^ = 2.8).

The standardised technical PMF sampler allows us to determine integrated deposition over the entire exposure period. The relationship between the results of standardised technical sampling using the PMF sampler and leaf pollen density measurements was experimentally evaluated based on parallel field measurements. The relationship between the PMF results and the mean daily pollen density on *Urtica* leaves was described by a calibration factor of 0.68, leading to an estimate of 234 n/cm^2^ for the mean daily leaf pollen density value over the flowering period at sites close to the pollen source (0.2 m) as average estimate taking into account the observed variability over time and between different sites under commercial cultivation and years. This value is 2.7 times higher than the EFSA Panel’s estimate of 85.56 n/cm^2^ for within-field mean daily leaf pollen density.

When determining adequate buffer isolation distances, it is important to define threshold levels of leaf pollen density that must not be exceeded. For their proposed isolation buffer distances of 20–30 m, the EFSA Panel calculated a maximum threshold value for mean leaf pollen density of 0.28 n/cm^2^ at 20 m and 0.01 n/cm^2^ at 30 m ([14] log_10_ *d* = 2346^−0.145*E*^ for distances *E* = 20 m and *E* = 30 m, where *d* is the leaf density in n/cm^2^ and *E* is distance in m). Considering the assessment based on the observed data of [2, 3], these thresholds can be consistently attained only at a kilometre or farther (1000 m; mean leaf pollen density with *Urtica* ‘realistic’ mean regression = 1.6 n/cm^2^; upper 95% confidence boundary for ‘worst case’ scenario = 69 n/cm^2^). Therefore Hofmann et al. [2, 3] recommended buffer isolation distances in the kilometre range instead of 20–30 m.

The comparison of leaf density values for *Urtica* in Fig. 1 confirms this conclusion for the actual EFSA Panel model [1, 4]. All three EFSA Panel scenarios (MR, DC, CO) generate considerably lower estimates than the one based on our observations, which covers the whole distance range from 0.2 to 4.42 km. The calculated threshold values at 20–30 m of the EFSA Panel scenarios can only be met beyond the kilometre range.

### Variability of leaf deposition data

Variability in leaf density matters because the dose–response curve for the toxicity of Bt pollen is highly non-linear, leading to considerably severer effects on sensitive Lepidoptera species [14, 36]. Despite acknowledging the relevance of this variability for exposure assessments in 2015 [4], the EFSA Panel focused only on mean values in 2016 [1]. Hofmann et al. [3] observed high variability in *Urtica* leaf pollen density values with a 95% upper confidence boundary of 2270 n/cm^2^ and maximum leaf density values of up to 13,802 n/cm^2^ close to the pollen source (0.2 m). The upper 95% confidence boundary indicating worst-case scenario based on the Hofmann et al. data follows the power function *d*_CS_ = 3949*S* ^−0.585^ (see Fig. 1). This exceeds the corresponding ‘conservative’ scenario of the EFSA Panel for a worst-case scenario (*d*_CO_ = 10.8*S* ^−0.585^) by more than 100 times.

## Conclusions

The interpretation of pollen deposition data and how to use them for risk assessments and risk management of Bt-maize differ widely between the approach of the EFSA Panel and the studies of Hofmann et al. The estimates of the EFSA Panel [1, 4] depend on an exposure model that is based only partially on observational data. To fill these data gaps, the model relies on the judgements of various experts, which leads to considerable uncertainty. We suggest that the estimates of leaf pollen deposition should be based rather on measured data, as shown in our previous integrated assessment study using standardised methods [3, 26]. This approach, using the most comprehensive dataset published in the literature so far, also has the advantage of representing realistic cultivation conditions. In our opinion, the validity of that study is not called into question by the arguments of the EFSA Panel [1].

Furthermore, the Hofmann et al. [2] data were incorporated inappropriately into the EFSA Panel model [1, 4] as depositions were greatly reduced based on new assumptions and with no consideration of measured variation.

A comparison of the three scenarios of the EFSA Panel model [1, 4] on the dose–distance relationship incorporating their own expert estimates shows that all three lie far below our empirical findings on *Urtica* leaf pollen density values. According to our results, the EFSA Panel underestimates the dose–distance relationship for the ‘most realistic’ scenario by a factor of 0.0138 (72-fold) compared with the empirically based results. For the ‘worst case’ scenario based on a 1:40 probability, the factor of underestimation is 0.00273 (365-fold).

According to our results, the conclusion of the EFSA GMO Panel [1, 4] “that the previous GMO Panel risk assessment conclusions and risk management recommendations on maize MON810, Bt11 and 1507 for cultivation remain valid and applicable” is, therefore, not scientifically justified.

In our opinion, to fulfil the requirements of an environmental risk assessment for Bt-maize cultivation aiming to protect sensitive species in nature reserve areas, deposition levels should be set in accordance with observed data. A realistic representation of the data must take into account the different sources of variability inherent to pollen release and deposition in the field. Thus, recommendations should incorporate data representing the variability of pollen deposition between sites and within sites, including the heterogeneous distribution between plants and on leaf surfaces, as detected in our field measurements.

According to our results, buffer isolation distances in the kilometre range, with specific environmental impact assessments in case of commercial cultivation of Bt-maize within this zone, are necessary to attain deposition levels that are associated with no more than a 0.5 to 1% mortality for highly sensitive butterfly species in the EU.

## Additional file

**Additional file 1.** Additional information.

## Abbreviations

EFSA: European Food Safety Authority
PMF: pollen mass filter, a passive aerosol sampler according to Association German Engineeers VDI Standard 4330-3/CEN TS 16817-1
PMO: a volumetric pollen monitor with omnidirectional inlet (TIEM technic GbR, Dortmund, Germany)
GM: genetically modified
GMO: genetically modified organism
NT: non-target
NTO: non-target organism
FFH areas: areas listed according to the EU FFH Habitats Directive (fauna–flora-habitats-Directive)
