# Erratum to: Reply to the EFSA (2016) on the relevance of recent publications (Hofmann et al. 2014, 2016) on environmental risk assessment and management of Bt-maize events (MON810, Bt11 and 1507)

**DOI:** 10.1186/s12302-017-0114-0

**Published:** 2017-04-26

**Authors:** Maren Kruse‑Plass, Frieder Hofmann, Ulrike Kuhn, Mathias Otto, Ulrich Schlechtriemen, Boris Schröder, Rudolf Vögel, Werner Wosniok

**Affiliations:** 1 TIEM Integrated Environmental Monitoring, Dortmund/Bremen, Germany; 2 Wölsauerhammer, Marktredwitz, Germany; 3 Ökologiebüro, Bremen, Germany; 4 Büro Kuhn, Bremen, Germany; 5 Federal Agency for Nature Conservation (BfN), Bonn, Germany; 6 Sachverständigenbüro, Dortmund, Germany; 7 Landscape Ecology and Environmental Systems Analysis, Institute of Geoecology, Technische Universität, Brunswick, Germany; 8 Berlin-Brandenburg Institute of Advanced Biodiversity Research (BBIB), Berlin, Germany; 9 Agency for Environment, Health and Consumer Protection, Eberswalde, Brandenburg Germany; 10 Institute of Statistics, University of Bremen, Bremen, Germany

## Erratum to: Environ Sci Eur (2017) 29:12 DOI 10.1186/s12302-017-0106-0

Upon publication of the original article [1], it was noticed that in the HTML version, two signs in the captions of Fig. 1 were the wrong way round.

The grey circle:  should denote ‘leaf pollen density data *Urtica* close to the pollen source indicating the variability and used for calibration (*n* = 836 measurement data, scattered around 0.2 m distance for displaying the variability close to source).’

The pink dotted line:  should denote ‘DC—‘direct comparison’ scenario EFSA panel model 2015’.

This has now been updated in the original HTML version of the article. This error arose due to our systems not processing the authors’ original correction request, therefore we, the publishers, apologise for this error and any inconvenience caused by it.
