# Supplementary material for: Reply to the EFSA (2016) on the relevance of recent publications (Hofmann et al. 2014, 2016) on environmental risk assessment and management of Bt-maize events (MON810, Bt11 and 1507)
Source: Environ Sci Eur. 2017 Mar 7;29(1):12. doi: 10.1186/s12302-017-0106-0 (PMC5340831; doi:10.1186/s12302-017-0106-0)

Supplements

**S1:** Exponential model of EU risk assessment compared with power model described in Hofmann et al.2014 (Figure 5)


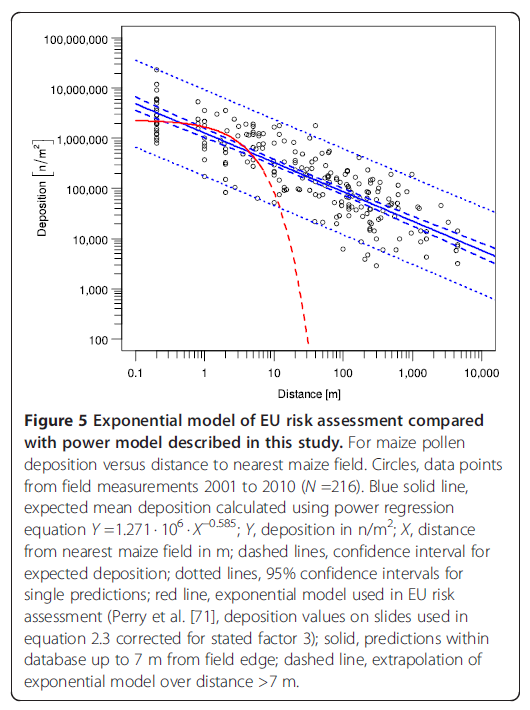


**S2**: The 8 uncertainty factors of EFSA 2015 (Table 2)


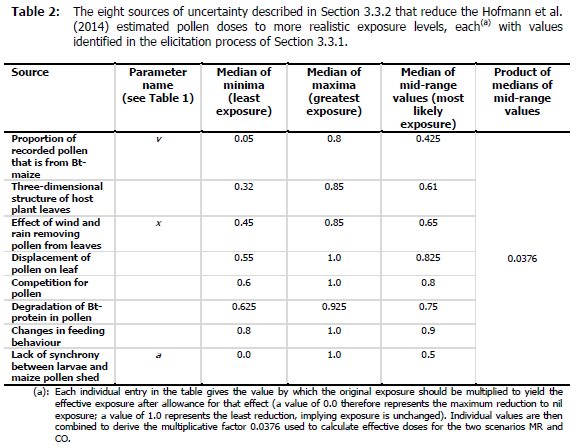


S3: Maize pollen deposition versus distance to the next maize field in Hofmann et al. 2014

S4: Standardized residuals for the regression in Hofmann et al. 2014

S5: Structured design: The structured design (last column) reflects best mean, variability and maximum peaks of the true leaf density distribution (column “Total”)


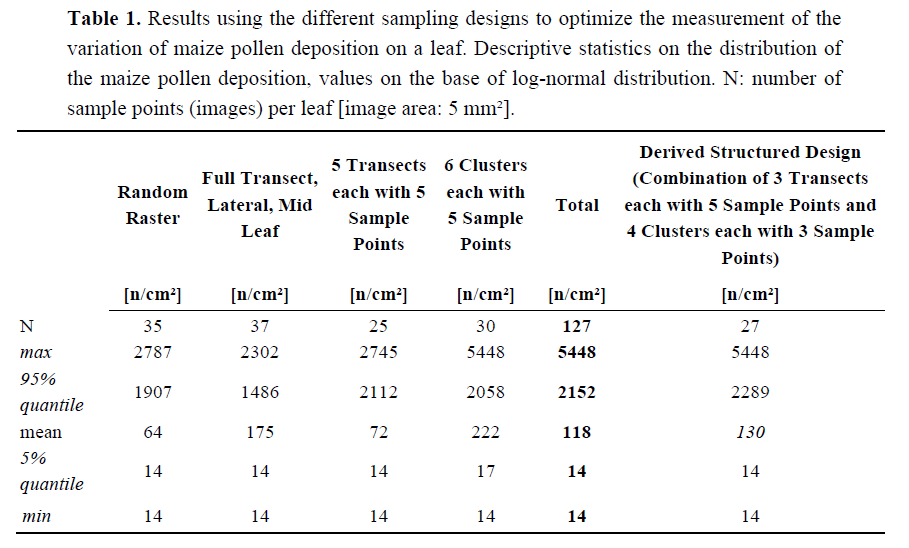


S6: Fig. 4, Hofmann et al. 2016


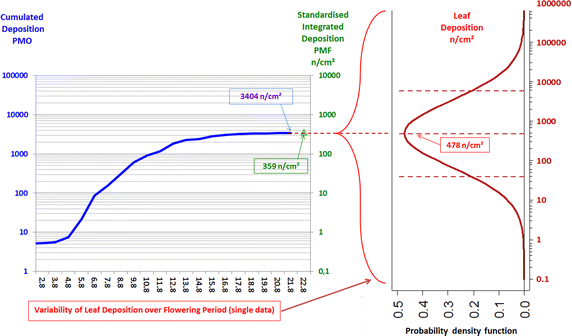


Fig. 4

Combining plant-specific leaf deposition and its variability with standardised measurements of pollen deposition. The blue line indicates cumulative maize pollen deposition, *D* _total_ (=cumulative daily deposition rate), measured with a PMO pollen monitor. The green values show standardised integrated deposition, *D* _S_, measured with a PMF passive sampler. The red curve shows the variability of plant-specific leaf deposition on maize leaves over the flowering period (log-normal distribution) with mean and 90 % confidence intervals.

S7: Variability of leaf density over the flowering period and standardized deposition
(Fig. 3 Hofmann et al. 2016)


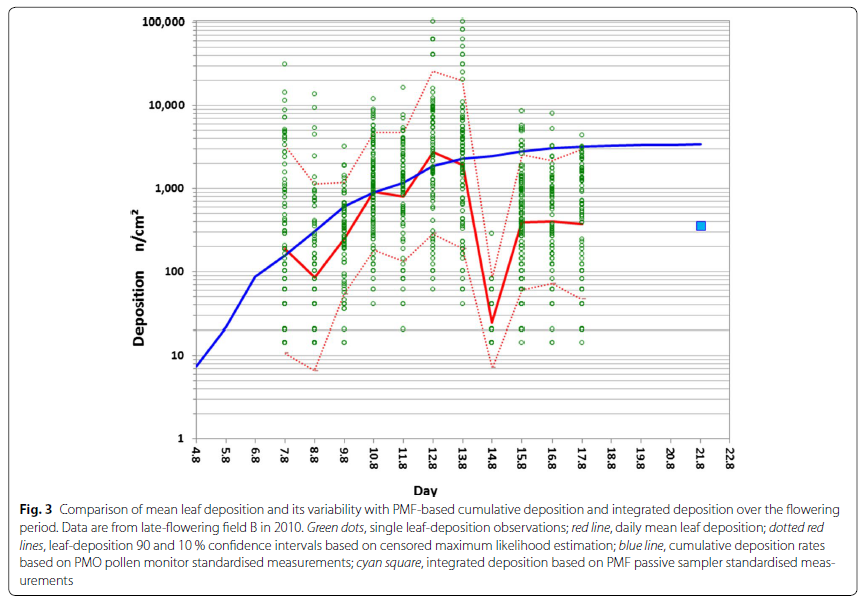

Supplement: Supplementary file 1 — Additional file 1. Additional information. [file 12302_2017_106_MOESM1_ESM.docx]
